# miR-186 affects the proliferation, invasion and migration of human gastric cancer by inhibition of Twist1

**DOI:** 10.18632/oncotarget.13182

**Published:** 2016-11-07

**Authors:** Chunhong Cao, Deguang Sun, Liang Zhang, Lei Song

**Affiliations:** ^1^ Department of General Surgery, The Second Affiliated Hospital of Dalian Medical University, Dalian, 116027 Liaoning, China

**Keywords:** miR-186, Twist1, GC

## Abstract

Recent evidence shows that miRNAs are dysregulated in a variety of cancers including gastric cancer (GC), and emerging as key oncogenes or tumor suppressors. In this study, qRT-PCR was used to analyze the expression of miR-186 in GC tissues and adjacent non-cancerous tissues, and then more in-vitro experiments were used to investigate the role of miR-186 in GC cells. Here, we identified miR-186 was generally down-regulated in GC tissues; however, Twist1 was generally up-regulated in GC tissues. Moreover, miR-186 and Twist1 were associated with larger tumor size and advanced clinical stage of GC. In-vitro experiments demonstrated that ectopic overexpression of miR-186 inhibited GC cell proliferation, invasion and migration; however, inhibited expression of miR-186 enhanced cell proliferation, invasion and migration. Furthermore, the luciferase reporter assay demonstrated Twist1 as a direct target of miR-186. Finally, over-expression of Twist1 abrogated inhibitory impact of miR-186 on cell proliferation, invasion and migration. In conclusion, miR-186 affects the proliferation, invasion and migration of human gastric cancer by inhibition of Twist1, and could be a tumor suppressor in GC development. Thus, miR-186 may be served as a candidate prognostic biomarker and target for new therapies in human gastric cancer.

## INTRODUCTION

Although there is an obvious decrease in the prevalence of gastric cancer, gastric cancer still acts as the second one in the most common malignancies in the real world, especially in China [1, 2]. To our pity, the five years overall survival rate of gastric cancer patients undergoing surgical operation remains to be not satisfying. To date, the common metastasis and recurrence have been reported as the main rationales, which affect the long-term survival of postoperative gastric cancer patients [3]. In addition, it is very difficult to diagnose gastric carcinoma at an early stage for some patients due to no clinical manifestations. Ergo, it is essential to figure out the pathogenesis and molecular mechanisms of gastric cancer to facilitate the clinical treatment of patients with GC.

In recent years, a large quantity of oncogenes and tumor suppressors has been reported to be involved in the regulation of the development of GC. In addition to putative protein-coding genes, non-coding miRNAs were reported as an extremely conserved RNA sequences, including 20–27 nucleotides. According to recent studies, miRNAs have the capacity of regulating the gene expression by targeting the 3′-untranslated regions (UTR) of related mRNAs in the development of different tumors [4, 5]. miRNAs can control the gene expression based on different levels, involving the modification of chromatin, gene transcription, proliferation, invasion and migration [6–8]. Increasing studies identified that miR-186 can be found to be down-/up-regulated in diverse cancer tissues. At the same time, it has been reported that miR-186 has a wide role in regulation of some functions involving suppression of cancer cell proliferation and inhibition of organ metastasis of different cancers. As reported, the expression of miR-186 was found to be down-regulated in some cancers, inducing prostate cancer, colorectal neuroendocrine tumors, ovarian cancer and non-small cell lung cancer [9–12]. However, little is known about the role of miR-186 in the development of GBM.

In view of the importance of miR-186 in the development of GC, we investigated miR-186 as a subject, and observed that miR-186 was obviously down-regulated in GC tissues as compared with their adjacent normal tissues using qRT-PCR analysis. Meanwhile, we also found that ectopic expression of miR-186 could affect cell proliferation, invasion and migration of gastric cancer partially via targeting the expression of Twist1.

## RESULTS

### The expression of miR-186 and Twist1 in GC samples

**Figure 1:**
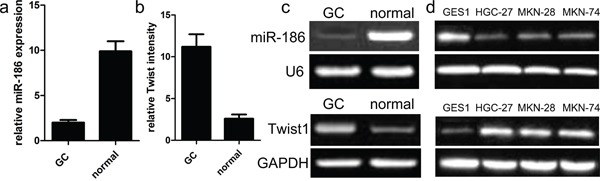
Relative miR-186 and Twist1 expression in GC tissues **a., b.** miR-186 and Twist1 expression levels in GC and normal tissues were analyzed using qRT-PCR. miR-186 and Twist1 expression were examined by qRT-PCR, and normalized to U6 and GAPDH expression (shown as ΔCT) *p<0.001, v.s. normal tissues, one-way ANOVA. **c.** Relative expression of miR-186 and Twist1 in human GC tissues compared with corresponding normal tissues. **d.** miR-186 and Twist1 expressions in human GC cell lines were compared with gastric epithelial mucosa cell line GES1 by qRT-PCR.

To figure out the significance of miR-186 and Twist1 in the development of gastric cancer, firstly, we detected and analyzed the expression of miR-186 and Twist1 in cancer samples of GC and their paired normal samples. In the present study, qRT-PCR analysis was applied to detect the expression of miR-186 in 90 cases of GC samples and their adjacent normal tissues according to histology. As shown in Figure 1a, we found that the expression of miR-186 was indeed reduced in tumor tissues as compared with that in their adjacent paired normal samples. Our findings also showed that the expression of Twist1 was indeed increased in cancerous tissues than in their adjacent normal tissues (Figure 1b). These findings indicated that miR-186 and Twist1 might be involved in the development of gastric cancer.

### The expression of miR-186 and Twist1 is correlated with clinical characteristics

As mentioned above, we further evaluated the relationship between miR-186 and clinicopathologic features of gastric cancer, all 90 cases of GC samples were divided into two groups according to the median of relative expression intensity of miR-186 in cancer samples, including low miR-186 expression group and high miR-186 expression group. The clinicopathological characteristics of 90 GC patients were summarized in Table 1. Notably, low miR-186 expression in GC was significantly correlated with advanced TNM stage, lymph node metastasis and tumor size (all p<0.000). However, miR-186 expression was not associated with other parameters such as gender and age et.al (Table 1). As for the relationship between Twist1 and clinicopathologic features of gastric cancer, we observed that high Twist1 expression in GC was significantly correlated with advanced TNM stage, lymph node metastasis and tumor size (all p<0.000). However, the expression of Twist1 was not associated with gender and age (Table 1).

**Table 1:** Correlations of miR-186 and Twist1 with clinicopathological indicators

Indicators	N	miR-186	*p* value	Twist1	*p* value
		Expression level		Expression level	
Age					
<56	45	2.2±0.2	0.158	11.6±2.9	0.563
≥56	45	1.9±1.4		11.3±1.9	
Gender					
male	60	1.8±1.3	0.120	11.1±1.1	0.266
female	30	2.2±0.7		10.7±2.3	
Tumor size					
<5cm	65	2.3±0.5	0.001	13.7±1.4	0.000
≥5cm	25	1.7±0.7		8.8±1.1	
Histology					
Well/moderate	59	2.5±0.6	0.000	7.9±1.2	0.000
Poor	31	1.5±0.8		13.3±1.7	
LN metastasis					
Yes	51	1.4±0.5	0.000	13.6±1.4	0.000
No	39	2.6±0.7		8.5±1.1	
TNM stage					
I-II	29	2.5±0.5	0.000	8.7±1.8	0.038
III-IV	61	1.6±0.7		14.2±1.4	

### Effect of miR-186 on GC cell proliferation

**Figure 2:**
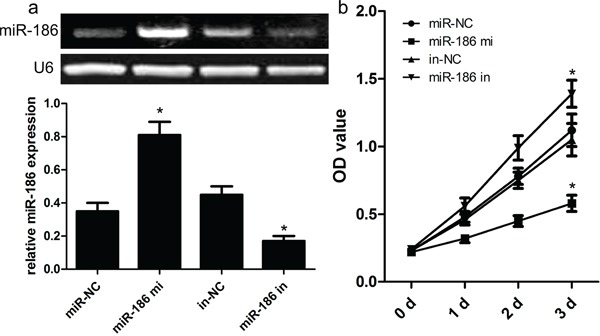
Effect of miR-186 on HGC-27 cell proliferation **a.** Relative expression levels of miR-186 in HGC-27 cells transfected with miR-186 mimics and inhibitors was tested by RT-PCR. **b.** MTT assays showed that miR-186 overexpression had an inhibitory effect on the growth of HGC-27 cells compared with their matched cells. *p<0.001, one-way ANOVA.

Mechanically, we continued to explore the function and role of miR-186 in the tumor biology of GC cell. At first, we carried out the qRT-PCR analysis to analyze the expression model of miR-186 in three kinds of human GC cell lines. As shown in Figure 1, the expression of miR-186 was significantly down-regulated in human GC cell lines HGC-27, MKN-28, and MKN-74, but the expression of miR-186 was not changed in the normal gastric epithelial mucosa cell line GES1. On the other hand, the expression of Twist1 was obviously up-regulated in human GC cell lines HGC-27, MKN-28, and MKN-74, but the expression of Twist1 was decreased in human gastric epithelial mucosa cell line GES1. To elucidate the biological role of miR-186 in the development of GC, miR-186 mimics and inhibitors were transfected into HGC-27 cells respectively. Afterwards, the qRT-PCR analysis was carried out at post-transfection 48 hours. Our findings revealed that the expression of miR-186 was significantly increased in miR-186 mimics-transfected HGC-27 cells, but not changed in inhibitor-transfected HGC-27 cells compared with their own controls (Figure 2a). Subsequently, MTT assays revealed that the overexpression of miR-186 exerted an inhibitory effect on the proliferation of HGC-27 cells as compared with miR-NC (Figure 2b), which suggested that miR-186 might participate in other biological processes of GC.

### miR-186 overexpression inhibits invasion and migration

**Figure 3:**
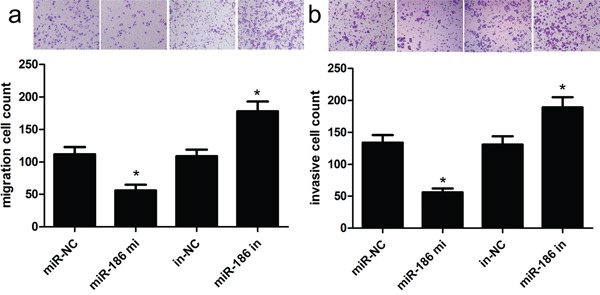
Overexpression of miR-186 could inhibit HGC-27 cell invasion and migration Transwell assays were used to investigate the changes in migratory **a.** and invasive **b.** abilities of HGC-27 cells. The number of invasive and migratory cells was significantly decreased due to miR-186 overexpression. *p<0.001, one-way ANOVA.

Next, we carried out transwell assays to evaluate the influence of miR-186 on GC cell invasion and migration. As shown in Figure 3, the overexpression of miR-186 inhibited the invasive ability of HGC-27 cells. Specifically, the number of invasive HGC-27 cells was obviously decreased as compared with their controls (p<0.001). As expected, the overexpression of miR-186 inhibited the migratory ability of HGC-27 cells. Specifically, the number of migratory HGC-27 cells was obviously decreased as compared with their controls (p<0.001). To further investigate the molecular mechanisms underlying miR-186-mediated proliferation, invasion and migration of GC cells, we used western blot analysis to detect related targets, and observed that overexpression of miR-186 reduced the expression of Twist1 protein. These findings mean that miR-186 could inhibit GC cell invasion and migration probably via controlling the expression of Twist1.

### Twist1 is a direct target of miR-186

**Figure 4:**
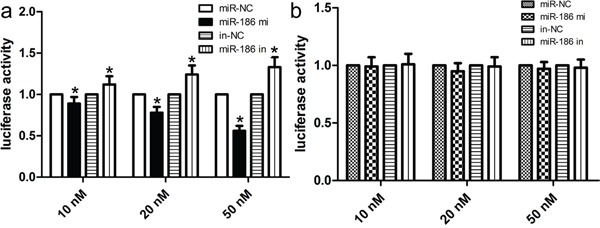
miR-186 was a direct transcriptional target of Twist1 in HGC-27 cells The miR-186 mimic inhibited the luciferase activity controlled by wild-type Twist1 −3′-UTR **a.** but did not affect the luciferase activity controlled by mutant Twist1 −3′-UTR **b.** in HGC-27 cells. Results shown are the mean ± SD of repeated independent experiments. *p<0.001, compared with control, one-way ANOVA.

To explore the molecular mechanisms by which miR-186 medicates the proliferation, invasion and migration, we determined a few candidate target genes of miR-186 using putative online databases. Of these candidates, Twist1 was chosen and exhibited the highest prediction scores because the sequence of Twist1 mRNA had the most complementary sequences with those of miR-186. After that, we performed a luciferase activity assay to identify whether Twist1 genes was indeed targeted by miR-186. In this work, luciferase vectors containing the wild type/mutation type 3′-UTR of Twist1 were constructed, and co-transfected along with the miR-186 mimic/inhibitor into cells. Our findings showed that the expression of miR-186 remarkably decreased the activity of wild type 3′-UTR of Twist1 in a dose-dependent fashion (p<0.001; Figure 4a), however the expression of miR-186 did not change the activity of mutant 3′-UTR of Twist1 (p>0.001; Figure 4b). Taken together, these findings suggested that Twist1 was a direct target for miR-186 in the development of GC.

### Up-regulated Twist1 expression attenuates miR-186-inhibited tumor biology

**Figure 5:**
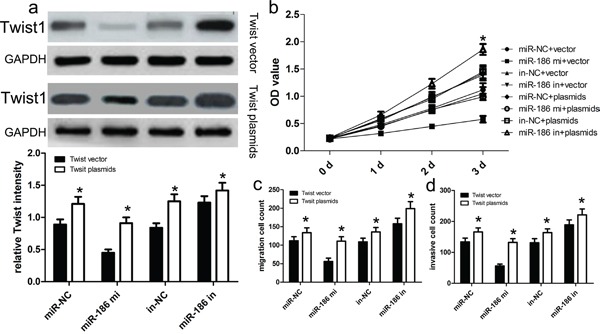
Twist1 overexpression reversed the inhibitory effect of miR-186 on HGC-27 cell proliferation, migration and invasion **a.** Western blot analysis showed that transfecting the cells with Twist1 plasmids could up-regulate Twist1 expression. The expression of Twist1 was normalized to that of GAPDH. **b.** The proliferation capacity of miR-186-overexpressing HGC-27 cells was partially improved when cells were transfected with Twist1 plasmids in comparison with control. **c., d.** The migration and invasion of miR-186-overexpressing HGC-27 cells were effectively improved when cells were transfected with Twist1 plasmids. *p<0.001, vs. vector, one-way ANOVA.

To further determine whether miR-186-dependent inhibition of GC cell proliferation, invasion and migration was indeed mediated by regulation of Twist1, we transfected cells with the Twist1 expression vector to restore Twist1 expression in HGC-27 cells (Figure 5a). We found that the restoration of Twist1 expression improved the miR-186-inhibited proliferation ability of HGC-27 cells (p<0.001; Figure 5b). At the same time, the restoration of Twist1 expression significantly abrogated miR-186-induced inhibition of HGC-27 cell migration and invasion (both p<0.001; Figure 5c and 5d).

## DISCUSSION

In recent decades, increasing reports have highlighted the molecular mechanisms and significance of the expression of miR-186 in different human tumors, such as lung cancer, gastric cancer [13–16]. Emerging evidence demonstrated that dysregulation of expression of miRNAs in gastric cancer might be thought as one of the leading factors in tumorigenesis, and miRNAs can be recommended as a predictive biomarker in the diagnosis of gastric cancer patients. Besides, Twist1 has been widely reported as a poor prognosis biomarker in diverse tumors [17–19]. Thus, identification of GC-related miRNAs will make for understanding of etiology of gastric cancer, and elucidation of their biological roles can facilitate the diagnosis and prognosis of gastric cancer patients. In this study, we found that the expression of miR-186 was generally down-regulated in GC samples, indicating that the expression of miR-186 might independently predict a poor prognosis in patients with gastric cancer. On the basis of cell functions, the overexpression of miR-186 is able to inhibit cell invasion and migration as well as cell proliferation. These findings suggest that miR-186 played an important role in regulation of GC cell proliferation, invasion and migration.

Subsequently, we selected a useful target Twist1 to explore the miR-186-Twist1 pathway. Firstly, Twist1 is a well-known transcription factor in the progression of the epithelial-mesenchymal transition (EMT). Twist1 has been reported to be involved in the de-differentiation of cancer cells, leading to cell invasiveness and migration. Other reports identified that Twist1 expression could enhance the tumor response to radiotherapy. However, the more molecular mechanisms of Twist1 involved in the development of gastric cancer remain unclear. In this work, we used the luciferase-based reporter assay and identified that miR-186 can bind a conservative sequence within the 3′-UTR of Twist1 to partially inhibit the expression of Twist1. After that, we transfected cells with the Twist1 expression vector to restore Twist1 expression in HGC-27 cells, and found that the restoration of Twist1 expression improved the miR-186-inhibited proliferation ability of HGC-27 cells. These findings indicate that Twist1 activated by down-regulation of miR-186 resulted in the proliferation, migration and invasion.

In oncogenesis, tumor-suppressing genes including miRNAs could be effectively silenced using epigenetic approaches [20–23]. In our study, we thought that miR-186 might be epigenetically repressed by histonedemethylase et al. Besides, Twist1 is identified as a target of miR-186, but Twist1 is not the only one that can be targeted by miR-186 for a single miRNA is more likely to regulate thousands or hundreds of mRNAs in oncogenesis. Therefore, it is essential to identify other potential novel targets of miR-186 or Twist1-related miRNAs, which will allow us to understand the deep molecular mechanisms underlying the development and progression of gastric cancer.

In conclusion, we for the first time demonstrated that miR-186 expression is down-regulated in gastric cancer, and overexpression of miR-186 inhibited cell proliferation, invasion and migration partially via regulation of Twist1 expression. Generally, this study provides a new idea that miR-186 may act as a novel target for early diagnosis and treatment of gastric cancer patients. However, the more detailed molecular mechanisms by which miR-186 promoted the development and progression of gastric cancer need further investigation.

## MATERIALS AND METHODS

### Tissue collection

90 paired GC and corresponding adjacent nontumorous samples were obtained from patients who underwent surgery at The Second Affiliated Hospital of Dalian Medical University between 2014 and 2015. All cases were confirmed as GC based on histopathological evaluation. The clinicopathological characteristics of the GC patients were summarized. No local or systemic treatment was conducted in these patients before surgery. All collected tissue samples were immediately snap-frozen in liquid nitrogen and stored at −80°C until required. Our study was approved by the Research Ethics Committee of The Second Affiliated Hospital of Dalian Medical University. Written informed consent was obtained from all patients.

### Cell culture

Human GC cell lines HGC-27, MKN-28, and MKN-74 as well as the immortalized human gastric epithelial mucosa cell line GES1 were purchased from the Cell Bank of Type Culture Collection of Chinese Academy of Sciences (Shanghai, China). All cell lines were cultured in Dulbecco's modified Eagle's medium supplemented with 10% fetal bovine serum, 100 U/ml penicillin and 100 μg/ml streptomycin (all purchased from Gibco Life Technologies, Grand Island, NY, USA). All cells were cultured at 37°C in a 5% CO2 atmosphere.

### RNA extraction and qRT-PCR assays

Total RNA was extracted from tissues or cells using Trizol reagent (Invitrogen, Carlsbad, CA). 1 μg total RNA was reverse transcribed to cDNA by using a reverse transcription kit for next qRT-PCR assays (Takara, Dalian, China). QRT-PCR assays were performed using SYBR Premix ExTaq II kit (Takara, Dalian China), and the results were normalized to the GAPDH expression level. All the PCR primers used in this study were shown as follows.

miR-186, forward: 5′-GCGGCGCAAAGAATTCT CCT-3′ and reverse: 5′-GTGCAGGGTCCGAGGT-3′;

U6, forward: 5′-CTCGCTTCGGCAGCACA-3′, and reverse: 5′-AACG CTTCACGAATTTGCGT-3′;

Twist1, forward: 5′-CGCCCCGCTCTTCTCC TCT-3′ and reverse: 5′-GACTGTCCATTTTCTCCT TCTCTG-3′

GAPDH, forward: 5′-GGGAGCCAAAAGGGTC AT-3′ and reverse: 5′-GAGTCCTTCCACGATACCAA-3′;

The qRT-PCR assays and data collection were performed on ABI 7500, and data were analyzed as threshold cycle values (ΔCt) and then converted to fold changes using the 2^−ΔΔCt^ method.

### Synthetic miRNA transfection

GC cell line HGC-27 was plated for approximately 24 h before transfection, miR-186 mimics, inhibitor and their respective miR-negative controls (Genepharma, Shanghai, China) were transfected using Lipofectamine™ 2000 (Invitrogen, USA) according to the manufacturer's procedures. Total RNA and protein were extracted at 48 h post-transfection and subjected to RT-qPCR, western blotting.

### Plasmid generation

The Twist1 sequence was synthesized and subcloned into the pcDNA3.1 vector (Invitrogen, Shanghai, China). Ectopic expression of Twist1 was achieved through pcDNA-Twist1 transfection, with an empty pcDNA vector used as a control. The expression levels of Twist1 were detected by qRT-PCR. Cells were grown in 6-well plates until confluent, then transfected with Lipofectamine 2000 (Invitrogen, Shanghai, China) according to the manufacturer's instructions. At 48 h post transfection, cells were harvested for qRT-PCR or western blot analysis.

### MTT assays

Cell viability was tested with a Cell Proliferation Reagent Kit I (MTT) (Roche Applied Science). The cells were grown in 96-well plates. Cell viability was assessed every 24h following the manufacturer's protocol. All experiments were performed in quadruplicate.

### Cell migration and invasion assays

Transwells (Corning, Tewksbury, MA, USA, 8.0-μm pores) were used to measure GC cell migration and invasion ability. For the migration assays, at 48 h post transfection, 3 × 10^4^cells in serum-free media were placed into the upper chamber of an insert (8-mm pore size; Millipore, Billerica, MA, USA). For the invasion assays, 1 × 10^5^ cells in serum-free medium were placed into the upper chamber of an insert coated with Matrigel. Medium containing 10% FBS was added to the lower chamber. After incubation for 24 h, the cells remaining on the upper membrane were removed with cotton wool. Cells that had migrated or invaded through the membrane were stained with methanol and 0.1% crystal violet, imaged, and counted using an IX71 inverted microscope (Olympus, Tokyo, Japan). Experiments were independently repeated three times.

### Western blot assay and antibodies

Cells protein lysates were separated by 10% SDS-polyacrylamide gel electrophoresis (SDS-PAGE), transferred to 0.22μm NC membranes (Sigma) and incubated with specific antibodies. Autoradiograms were quantified by densitometry (Quantity One software; Bio-Rad). GAPDH antibody was used as control, and Anti-Twist1 (1:1000) were purchased from Cell Signaling Technology, Inc (CST).

### Luciferase reporter assay

To construct a luciferase reporter vector, Twist1 3′-UTR fragment containing putative binding sites for miR-186 was amplified by PCR, and then the PCR product was subcloned downstream of the luciferase gene in the pLUC Luciferase vector (Ruibo, Guangzhou, China) and named Twist1 −3′-UTRWT. For the mutated construct, cells grown in 96-well plate were transfected with 100 ng of Twist1 −3′UTR-Wt or Twist1 −3′UTR-Mut, using the Lipofectamie 3000 (Invitrogen, USA). After 72 h of transfection, luciferase activity was assessed according to the Dual-Luciferase Reporter Assay protocol (Promega, Madison, WI). Each experiment was repeated in triplicates.

### Statistical analysis

The Students t test (2 tailed), one-way ANOVA, and Mann-Whitney U test were conducted to analyze these data by SPSS 16.0 software. P values less than 0.05 were considered significant.
